# Olfactory threshold and odor discrimination ability in children – evaluation of a modified “Sniffin’ Sticks” test

**DOI:** 10.1038/s41598-017-01465-1

**Published:** 2017-05-16

**Authors:** Janine Gellrich, Carolin Stetzler, Anna Oleszkiewicz, Thomas Hummel, Valentin A. Schriever

**Affiliations:** 1 Abteilung Neuropädiatrie, Medizinische Fakultät Carl Gustav Carus, Technische Universität Dresden, Dresden, Germany; 2 Smell & Taste Clinic, Department of Otorhinolaryngology, “Technische Universität Dresden”, Dresden, Germany; 3 Institute of Psychology, University of Wroclaw, Wroclaw, Poland

## Abstract

The clinical diagnostics of olfactory dysfunction in children turns out to be challenging due to low attention span, insufficient linguistic development and lack of odor experiences. Several smell tests have been developed for adults. Most of these examinations take a relatively long time and require a high level of concentration. Therefore, the aim of the current study was to evaluate an odor discrimination and olfactory threshold test using the frequently used “Sniffin’ Sticks” in children and adolescents in a simplified two-alternative-forced-choice version (2AFC) and compare it to the original three-alternative-forced-choice test (3AFC). One-hundred-twenty-one healthy participants aged between 5 and 17 years took part in this study. Within each of the two sessions participants underwent olfactory testing using the modified 2AFC as well as the standard 3AFC method. A better test-retest reliability was achieved using the original 3AFC method compared to the modified 2AFC. This was true for the odor discrimination as well as the olfactory threshold. Age had a significant influence on both tests, which should be considered when testing young children. We discuss these findings with relation to the existing norms and recommend using the 3AFC version due to a better test-retest reliability to measure olfactory function in children.

## Introduction

Information about the prevalence of olfactory dysfunction in children is rare and numbers reported in the literature vary greatly. Due to neurodegenerative disease and cumulative loss of the olfactory epithelium caused by repeated infections, olfactory disorders are more common in adults than in children^1^. Nevertheless, hereditary anosmia and olfactory dysfunction due to traumatic brain injury, obesity or anorexia are only a few examples of etiologies for smell disturbance in childhood^2–5^.

The clinical diagnostics of olfactory dysfunction turns out to be challenging due to low attention span, linguistic development and lack of odor experience^6–8^. Several smell tests have been developed for adults. The most commonly used tests are: the “Sniffin’ Sticks” test battery (Hummel *et al*.^9^; Hummel *et al*.^10^; Kobal *et al*.^11^), the University of Pennsylvania Smell Identification Test (UPSIT) and the Cross Cultural Smell Identification Test (CCSIT)^12, 13^. Most of the tests designed for adults take relatively long time to perform and require high level of concentration during the testing procedure. Therefore, the testing procedure might be especially difficult for children, who become fatigued more easily and have shorter attention span than adults, that may result in higher randomness in their responses. Moreover, former reports show, that among children the odor identification score might depend on their verbal skills^6, 8, 14^.

To overcome these challenges, several olfactory testing in children have been developed. These attempts mainly focused on odor identification abilities, e.g., “Sniffin’ Kids” test with odors, which are more familiar to children^15^; a “Scratch and Sniff” card system for children^8^; a game-like identification test called “The Smell Wheel”^16^ or an olfactory assessment using the NIH Toolbox^17^. Additionally The Lyon Clinical Olfactory Test (LCOT) enables measurement of identification abilities, olfactory threshold and supra-threshold^6^. Especially to overcome the problem of insufficient verbal development in children a match-to-sample odor discrimination test for children was developed^18^. Within this test three odor stimuli are presented in a form of microencapsulated plastic cards (probe, match, distractor). Despite its utility, the test has rarely been used.

The aim of the current study was to develop a method suitable for the evaluation of an odor discrimination and olfactory threshold test in children by simplifying the frequently used “Sniffin’ Sticks” into a two-alternative-forced-choice test (2AFC) and compare it to the original three-alternative-forced-choice test (3AFC). Also, to exclude the bias resulting from variance in verbal development in children, we refrained from using the odor identification subtest (present in the original version of the “Sniffin’ Sticks”) and only examined the olfactory threshold and odor discrimination subtests.

## Results

Analyses were performed using IBM SPSS 22.0 (SPSS Inc., Chicago, IL, USA) software with significance-level set to α = 0.05. We performed two separate linear mixed models for threshold and discrimination scores. Within each model we included participants’ sex (male vs female), age group (5–8 vs 9–11 vs 12–14 vs 15–17), ordinal number of the session (first vs second) and the type of choice (2AFC vs 3AFC) as fixed factors. Number of days between the sessions was treated as a covariate.

### Olfactory threshold

**Figure 1:**
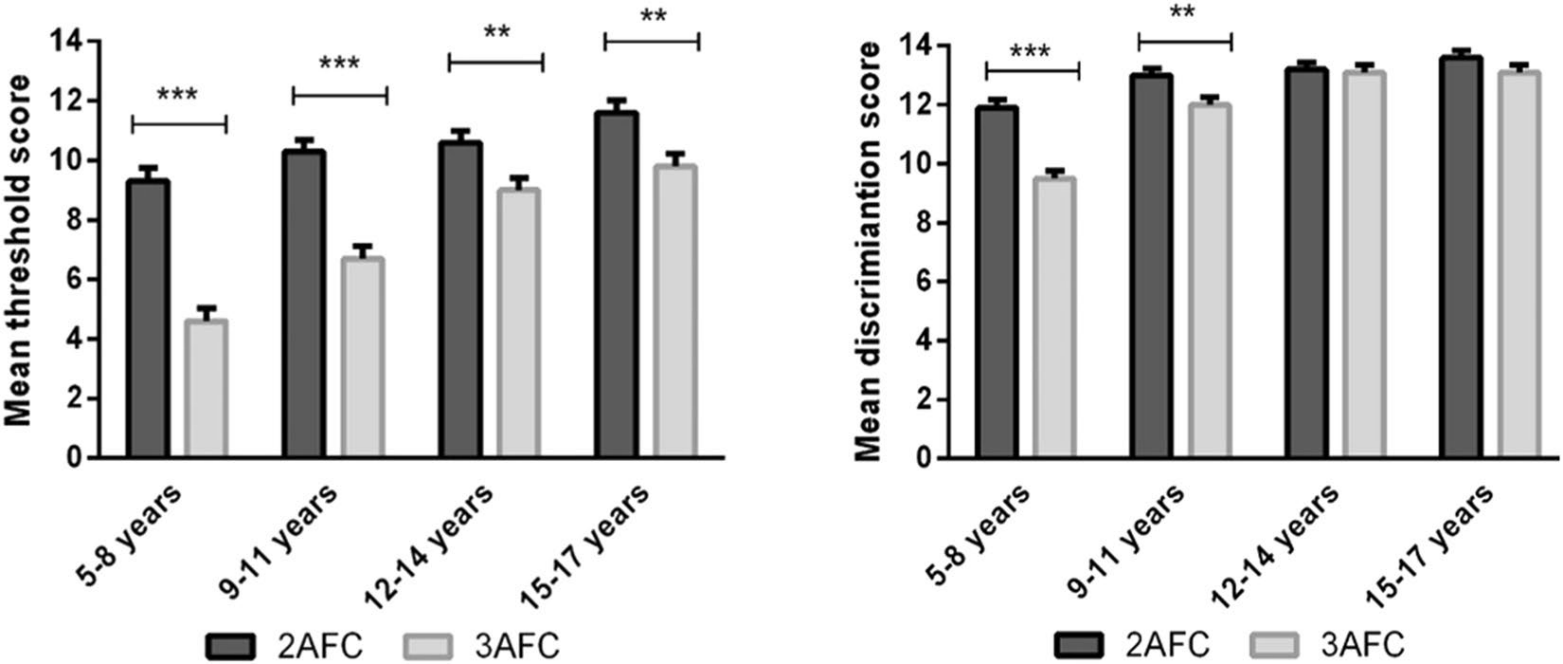
Olfactory threshold (1**a**) and odor discrimination (1**b**): mean scores separately for the 2AFC version and the 3AFC task across age groups. Error-bars indicated standard errors of means, *p < 0.05, **p < 0.01, ***p < 0.001. For detailed results see Table 1.

A fully factorial model revealed a significant main effect of age, *F*(3,417) = 29.5, *p* < 0.001, with pairwise comparisons indicating an increase in threshold scores with age (see: Table 1). Further, we found a significant main effect of the type of choice, *F*(1,417) = 99.8, *p* < 0.001, with pairwise comparisons showing that 2AFC (*M* = 10.4 ± 0.21) produced significantly higher threshold scores as compared to 3AFC (*M* = 7.5 ± 0.21). Interestingly, we found an interaction effect between the type of choice and age group, *F*(3,417) = 6.2, *p* < 0.001. Pairwise comparisons showed, that higher threshold results were obtained with 2AFC comparing to 3 AFC within each age group, however this difference was shrinking with age (all *p*s < 0.05 see Fig. 1). An interaction effect between age and sex was observed *F*(*3*,*417*) = *2.9*, *p* = *0.033*, but the pairwise comparisons showing only a significant effect for the fourth age group (p < 0.05) with female (M = 11.3 ± 0.41) outperform male (M = 10.0 ± 0.42).

**Table 1:** Scores of age groups measuring olfactory threshold and odor discrimination with 2AFC and 3AFC method (means ± standard error; n: number of subjects).

Age group	2AFC		3AFC	
	Olfactory threshold	Odor discrimination	Olfactory threshold	Odor discrimination
1 (5 to 8 years)	9.3 ± 0.45	11.9 ± 0.27	4.6 ± 0.43	9.5 ± 0.26
n	23	23	25	25
2 (9 to 11 years)	10.3 ± 0.39	13.0 ± 0.23	6.7 ± 0.42	12.0 ± 0.25
n	29	29	26	26
3 (12 to 14 years)	10.6 ± 0.39	13.2 ± 0.23	9.0 ± 0.41	13.1 ± 0.24
n	30	30	28	28
4 (15 to 17 years)	11.6 ± 0.41	13.6 ± 0.24	9.8 ± 0.42	13.1 ± 0.25
n	28	28	27	27

Olfactory threshold scores in phenyl ethyl alcohol dilution steps; odor discrimination scores in number correctly performed tasks.

### Odor discrimination

The model revealed a significant main effect of age, *F*(3,417) = 44.7 *p* < 0.001, with pairwise comparisons indicating an increase in discrimination scores with age (see: Table 1). We also found a significant main effect of the type of choice, *F*(1,417) = 34.4, *p* < 0.001, with pairwise comparisons showing that 2AFC (*M* = 12.9 ± 0.12) lead to significantly higher discrimination scores compared to 3AFC (*M* = 11.9 ± 0.12). In contrast to the first model, we found a significant main effect of participants’ sex, *F*(1,417) = 15.0, *p* < 0.001. Pairwise comparisons showed, that overall females scored significantly higher (*M* = 12.8 ± 0.12) than males (*M* = 12.1 ± 0.12). We also observed an interaction effect between age and sex, *F*(3,417) = 5.3, *p* = 0.001. Pairwise comparisons indicate that the significant difference between males and females could only be observed in groups aged between 5–8 and 12–14 years (*p*s < 0.05). Similarly to the first model, we found an interaction effect between the type of choice and age group, *F*(3,417) = 7.0, *p* < 0.001. Pairwise comparisons showed, that significantly higher discrimination results were obtained with 2AFC comparing to 3AFC among children aged 5–11(all ps <0.05), but for older children the difference was not significant (see Fig. 1).

### Reliability analysis

To verify the reliability of the measurement methods we calculated Pearson’s correlations for scores obtained across two sessions. For threshold testing between-session reliability of 3AFC was *r* = 0.620, *p* < 0.001, wherein for 2AFC it was r = 0.260, p = 0.006. For discrimination scores 3AFC reliability was *r* = 0.669, *p* < 0.001 and 2AFC was *r* = 0.514, *p* < 0.001. We used Fischer transformation to build confidence intervals for the correlation coefficients and found a significant difference between reliability coefficients for olfactory threshold *p* < 0.001 as well as for the odor discrimination *p* < 0.05. Detailed reliability coefficients across the age groups can be found in Table 2.

**Table 2:** Correlation coefficients and significance of age groups between first and second testing (n: number of subjects, r: correlation coefficient, p: level of significance).

Age group	2AFC		3AFC	
	Olfactory threshold	Odor discrimination	Olfactory threshold	Odor discrimination
1 (5 to 8 years)	r = 0.08 p = 0.72	r = 0.62 p < 0.01	r = 0.39 p = 0.06	r = 0.63 p = 0.001
n	23	23	25	25
2 (9 to 11 years)	r = 0.37 p < 0.05	r = 0.35 p = 0.07	r = 0.52 p < 0.01	r = 0.545 p < 0.05
n	29	29	26	26
3 (12 to 14 years)	r = 0.12 p = 0.54	r = 0.59 p = 0.001	r = 0.20 p = 0.30	r = 0.46 p < 0.05
n	30	30	28	28
4 (15 to 17 years)	r = 0.45 p < 0.05	r = 0.36 p = 0.06	r = 0.64 p < 0.001	r = 0.37 p = 0.06
n	28	28	27	27

Olfactory threshold scores in phenyl ethyl alcohol dilution steps; odor discrimination scores in number correctly performed tasks.

## Discussion

In the current study we evaluated a modified “Sniffin’ Sticks” test for olfactory threshold and odor discrimination. Results show that the 2AFC method yielded higher scores for both olfactory threshold and odor discrimination than the 3AFC method, which was especially pronounced in younger children. However, the reliability analysis disqualified the use of the simplified version of the “Sniffin’ Sticks” threshold and discrimination subtest.

The question, which n-AFC method is more suitable for sensory and cognitive tests, is not only focused on olfactory perception research. For example, there is a version of threshold-audiometry^19^, which is applied using  a 2AFC. A correct performance of 70.7% is obtained. A study from 1990 displayed that the same test using a 3AFC or 4AFC method would yield superior results but may make the task too complicated and influenced by short-term memory^20^. In the olfactory perception research the 3AFC method proved to be useful and is evaluated for the use of the “Sniffin’ Sticks” (r = 0.61)^9^. An olfactory test developed in 1988^21^ used the 2AFC method but showed lower test-retest reliability than the 3AFC method (r = 0.49)^22^. It can be assumed that the reliability of a test is not only influenced by the n-AFC procedure but is dependent on the sensory modality.

Comparison of the olfactory scores measured within the current study with the 3AFC paradigm are in line with those reported for the “Sniffin’ Sticks” normative data^10^. For children between 5 to 15 years normative data present a mean score of 6.59 ± 2.23 points for olfactory threshold and 12.32 ± 1.7 points for odor discrimination. We observed a mean threshold score of 7.5 ± 0.21 points and a mean odor discrimination score of 11.9 ± 0.12 points for children between age 5 and 17 years. The slightly higher mean scores observed in the current study might result from the difference in participants’ age, because in our sample we included older participants.

The 2AFC method resulted in higher scores in the olfactory threshold as well as in the odor discrimination test, most likely due to a higher likelihood of correct guesses (50% chance instead of the usual 33%). Former reports^23^ also used the 2AFC method with the “Sniffin’ Sticks” in order to measure the olfactory threshold and odor discrimination in children, however, until now the reliability of the 2AFC method with comparison to 3AFC method have not been reported.

Similar to the presented simplified version of the “Sniffin’ Sticks”, the Connecticut Chemosensory Clinical Research Center (CCCRC) also contains an olfactory threshold test using a 2AFC procedure, but the test is meant for adults^21^. The test-retest reliability of CCCRC shows a correlation coefficient of *r* = 0.49^22^ while we observed even lower reliability (r = 0.26, p = 0.006). One possible explanation for the difference in reliability could be the fact that the CCCRC was designed to measure olfactory function in adults, but not in children. In fact, we observed a threshold tests reliability of *r* = 0.45 for the oldest age group (15 to 17 years) in our sample what is much closer to the one reported for the CCCRC^22^ than the overall threshold reliability coefficient (*r* = 0.26). Further, the 3AFC method for threshold testing shows higher reliability coefficients than 2AFC method across all age groups.

In a study assessing extended version of the “Sniffin’ Sticks” the reliability of the original test reached *r* = 0.92, whereas reliability coefficient for the discrimination test was *r* = 0.69^24^ It can be concluded, that the reliability in olfactory threshold testing increases with the complexity of the method. While our correlation coefficient for 3AFC in discrimination is quite similar to the values reported earlier^24^, the score of olfactory threshold differs more (3AFC threshold r = 0.62, p = 0.001) but increase with age. Especially noticeable in our results is the low correlation score for olfactory threshold for the third age group in both test versions, which is not in line with the other measured age groups. Beside the fact that the number of participants is fairly small in each age group (n 23–30 participants) no convincing explanation could be found why children between an age of 12 and 14 scored lower on olfactory testing than both younger and older participants.

In the study concerning the development of olfactory ability in children aged between 3 to 12 years^6^ olfactory threshold and odor identification were investigated by means of the Lyon Clinical Olfactory Test. This tool is normally used to measure the olfactory function of adults. Results show that the school grade level has an influence on olfactory threshold scores. The authors hypothesized that the increase of olfactory threshold scores with age could be explained with a higher ability to concentrate and an increasing short-term memory^6^. Our results indicate that especially age has a significant influence on the olfactory threshold score across all age groups. Especially for the 3AFC method we observed a steep rise of olfactory scores while the 2AFC version shows only a slight increase for each age group, which is in line with former reports^23^.

This could arise the question if especially the olfactory threshold test of the “Sniffin’ Sticks” battery is suitable for the use in younger children: The “Sniffin’ Sticks” test has already been evaluated^10, 23^ and most children enjoy this game-like smell test. The precondition for the appropriate implementation of each psychophysical test is the understanding of the task. The cognitive development is intraindividual, so that some children might understand the procedure of the “Sniffin‘ Sticks” test by an age of 3 or 4 years while a little group of children at the age of 6 years is still not able to follow the instructions (e.g. exclusion of one participant in this study by failing to understand the task)^23^. Therefore, results of younger children need to be interpreted with caution. If children of younger age score high results it reduces the likelihood of an olfactory disorder. In our study the children of the first age group (5–8 years) scored lower with the 3AFC method than with the 2AFC method. Additionally, the 3AFC version showed a steeper rise to the next age group than the 2AFC. But even in this young children the between-session-reliability scored higher for 3AFC method than 2AFC.

Other studies examined even younger children starting at an age of 3 years^17, 23, 25, 26^. Some of them displayed difficulties like small sample size, incomplete measures^23^ or unfamiliarity with the odors in the identification task^17, 26^ while other results might be influenced through the instructions of parents^25^. Most of them showed poor results for children under an age of 5 years. This is the reason why we did not include children younger than 5 years in our study.

A possible limitation of the current study results from the fact that the children need to be blind-folded for the olfactory threshold and odor discrimination task and have to rely only on verbal communication during the tests. This might frighten especially younger children and could inhibit a strict concentration on the current task.

The 2AFC method presented in this study produces higher threshold and discrimination scores as the common 3AFC method, but at the same time cannot be considered as a reliable tool for studies involving children. The 3AFC version should be recommend measuring the olfactory function in children. Further investigations are recommended to adapt an odor discrimination and olfactory threshold test to children and control the influence of age and cognitive abilities but still keeping the test highly reliable. One possible starting point could be to analyze whether some odors of the discrimination task are more easily to distinguish for children than others to make these test less complex.

## Material and Methods

The study received the approval of the local Ethics Committee of the Medical Faculty of the Technical University of Dresden (EK 150042014). The study was conducted in accordance to the Declaration of Helsinki on Biomedical Studies Involving Human Subjects.

### Participants

A total of 121 healthy children between an age of 5 and 17 years participated in the study. Children and their parents/legal guardians did not report any disease, which could potentially cause olfactory dysfunctions. Nevertheless, we excluded two participants: the first (aged 6 years) failed to understand the task and the second (aged 16 years) was excluded due to the symptoms of fatigue and falling asleep during the second session of examination. The remaining sample consists of 119 children. Outliers were excluded with a cut off of two standard deviations for the 2AFC and 3AFC separately. Therefore, the final sample of the 2AFC method consists of 106 children (51 female) with an average age of 11.7 ± 3.6 years while 110 children (57 female) with an average age of 11.6 ± 3.7 years of the 3AFC method. In accordance with former reports on olfactory testing in children and adolescents, the participants were divided into four age groups: 1) (5–8; 2) (9–11; 3) (12–14; 4) 15–17 years^7^.

### Procedure

The purpose and procedure of this study was explained to the parents/legal guardians and children older than eight years, both verbally and in writing. Children younger than eight years received only a verbal explanation of the study. Written informed consent was obtained from parents/legal guardian prior to inclusion in the study. Every child voluntarily agreed to participate in the study.

We used the olfactory threshold and the odor discrimination subtests of the validated “Sniffin’ Sticks” battery^11^. Odorants were presented in felt-tip pens (“Sniffin’ Sticks”, Burghart GmbH, Wedel, Germany). For the presentation of the odors the cap of the pens was removed by the examiner for approximately 3 seconds and the tip of the pen was positioned approximately 2 cm under the nostrils.

Instead of pigment the pens for threshold testing were filed with phenylethylalcohol (flower or rose like odor) in dilution with propylene glycol. In the original version 16 triplets of pens were presented in a staircase procedure starting with the highest dilution (4%). One pen of the triplet contained diluted phenylethylalcohol while the other pens were only filed with propylene glycol. The interval between the presentation of pens of one triplet was 3 seconds. Between each triplet was a pause of 20 seconds. To prevent a visual identification of the odor containing pen the subjects were blindfolded. Using the 3AFC paradigm the participant had to decide which of the three pens contains the odor. Two successful odor-pen identifications or one false identification induced the next higher or lower staircase step. This procedure was repeated until seven reversal points had been obtained. The average of the last four reversal points revealed the individual threshold of the subject. A maximum score of 16 points can be achieved on the olfactory threshold test.

To fit the olfactory threshold test for the use in children we modified it in a way that instead of one target and two distractor pens (3AFC) we only used one distractor and one target (2AFC). The concentrations and staircase procedure remained the same as in the original version.

Beside the olfactory threshold the odor discrimination test was also administered. Again 16 triplets of pens were used with two pens containing the same odor and one having a different smell. In a 3AFC paradigm the participant had to identify the pen with the different smell. To prevent the visual identification of the target the subjects were blindfolded. A repetition of a triplet was not allowed. Between each of the triplets a pause of minimum 30 seconds was taken. The maximum score achievable on the odor discrimination test is 16 points.

For the study we also changed the 3 AFC paradigm of the odor discrimination subtest into a 2AFC version. A pair of “Sniffin’ Sticks” was presented to the participants – either having the same or different odors. Participants’ task was to decide whether the two “Sniffin’ Sticks” had the same odor or not. The number of “Sniffin’ Sticks” with the same (n = 8) and different (n = 8) odors was balanced.

The results obtained with the modified tests were compared to the results obtained with the standard “Sniffin’ Sticks” tests using the 3AFC method.

All children participated in two sessions, each lasting approximately one hour. During the first session participants underwent olfactory testing using the modified 2AFC as well as the standard 3AFC method. The session always started with the olfactory threshold test being followed by the odor discrimination test. The order of method (2AFC, 3AFC) was randomized between the participants. In the second session the tests were repeated in the same order to investigate the reliability of the methods. The sessions took place on separate days with a minimum interval of two days (mean 9.6 ± 8.8 days).
